# Temperature and the vertical movements of oceanic whitetip sharks, *Carcharhinus longimanus*

**DOI:** 10.1038/s41598-018-26485-3

**Published:** 2018-05-29

**Authors:** Samantha Andrzejaczek, Adrian C. Gleiss, Lance K. B. Jordan, Charitha B. Pattiaratchi, Lucy A. Howey, Edward J. Brooks, Mark G. Meekan

**Affiliations:** 1 Ocean Graduate School & The UWA Oceans Institute, The University of Western Australia, Crawley, WA 6009 Australia; 2 The Australian Institute of Marine Science, Crawley, WA 6009 Australia; 3 Centre for Sustainable Aquatic Ecosystems, Harry Butler Institute, Murdoch University, Murdoch, WA 6150 Australia; 4 Microwave Telemetry, Inc., Columbia, Maryland 21045 USA; 5 Shark Research and Conservation Program, The Cape Eleuthera Institute, Eleuthera, Bahamas

## Abstract

Large-bodied pelagic ectotherms such as sharks need to maintain internal temperatures within a favourable range in order to maximise performance and be cost-efficient foragers. This implies that behavioural thermoregulation should be a key feature of the movements of these animals, although field evidence is limited. We used depth and temperature archives from pop-up satellite tags to investigate the role of temperature in driving vertical movements of 16 oceanic whitetip sharks, *Carcharhinus longimanus*, (OWTs). Spectral analysis, linear mixed modelling, segmented regression and multivariate techniques were used to examine the effect of mean sea surface temperature (SST) and mixed layer depth on vertical movements. OWTs continually oscillated throughout the upper 200 m of the water column. In summer when the water column was stratified with high SSTs, oscillations increased in amplitude and cycle length and sharks reduced the time spent in the upper 50 m. In winter when the water column was cooler and well-mixed, oscillations decreased in amplitude and cycle length and sharks frequently occupied the upper 50 m. SSTs of 28 ^o^C marked a distinct change in vertical movements and the onset of thermoregulation strategies. Our results have implications for the ecology of these animals in a warming ocean.

## Introduction

Knowledge of how animals respond to the physical environment is vital for management strategies and prediction of how climate change will impact their ecology^1^. Water temperature is arguably the most influential physical driver governing the movements of marine animals, particularly for ectotherms that cannot internally regulate their body temperature^2^. As all major physiological processes are sensitive to changes in body temperature, the physiological performance of ectotherms is directly related to the temperature of the external environment^2^. Thus, in order to maximize performance, these animals must use behavioural strategies to thermoregulate by moving through their habitat in a manner as to maintain optimal body temperatures whenever possible^3^.

**Figure 1:**
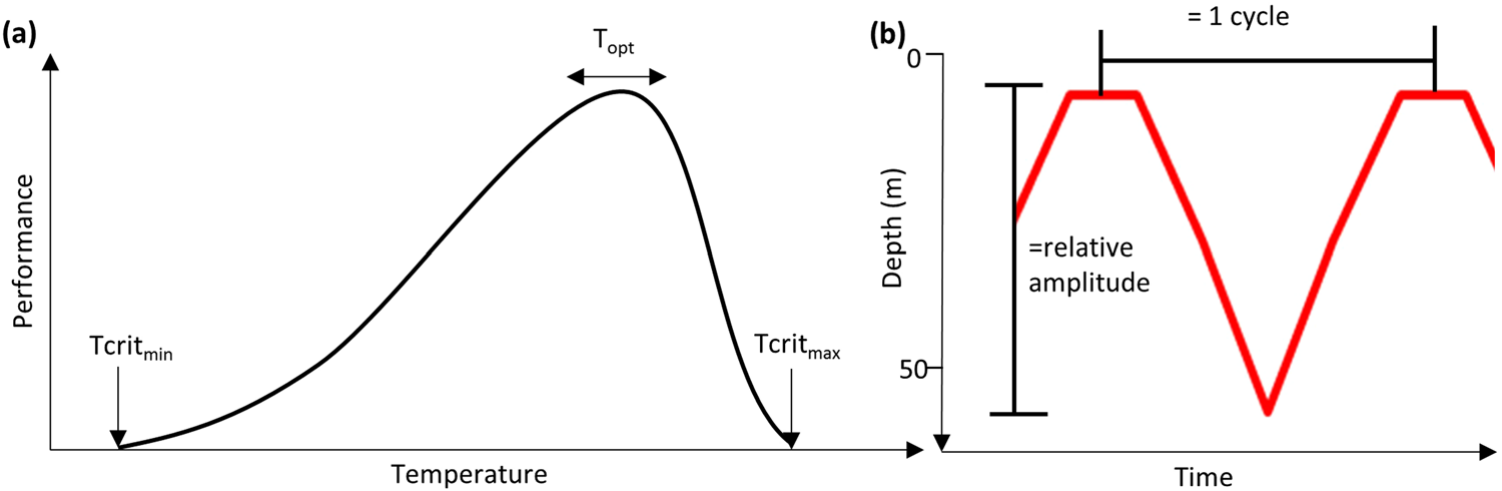
Schematic diagrams of (**a**) hypothetical performance curve of an ectotherm adapted from Huey and Stevenson (1979). The curve describes the temperature for which performance is optimised (T_opt_) and the critical minimum and maximum temperatures (Tcrit_min_ and Tcrit_max_) at which an organism can survive; and (**b**) the cyclic characteristics of oscillatory movements: mean cycle length and cycle amplitude extracted from a continuous wavelet transformation on depth data.

Thermal performance curves^4^ provide a theoretical basis for an understanding of this behaviour. These curves describe how body temperature influences physiological performance and are bounded by the minimum and maximum critical temperatures at which an organism can survive, and the temperatures at which performance is optimized (Fig. 1a). The curve is typically skewed so that performance gradually rises from a critical minimum temperature up to an optimum, before sharply dropping to a critical maximum. As a result, physiological performance is impacted more by an increase in temperature above optimum than by an equivalent decrease in temperature below optimum^5^. For terrestrial ectotherms, a global analysis has shown that the primary thermal challenge is to avoid overheating, especially in tropical and desert areas^6^. For this reason, description of behavioural strategies used as a response to temperature is particularly important in the context of warming global climate^6^.

Sharks and other pelagic fishes that inhabit epipelagic tropical environments could be considered to be the marine counterparts of these terrestrial ectotherms. Similar to deserts, tropical waters are warm and have low rates of primary production, so that these marine predators have high metabolic rates that must be maintained within an oligotrophic ecosystem where prey is sparsely distributed. In order to survive in these environments, ectotherms need to be cost-efficient foragers^7,8^ and must keep their body temperature within an optimal range. Large marine ectotherms are therefore likely to utilize movements throughout the water column in order to maintain a thermal range within the bounds required for survival (Fig. 1a). Given the typical shape of thermal performance curves, we predict that these movements will be most critical near upper limits of temperatures, where sharp breakpoints in vertical movement behaviours may become evident as animals seek to maintain body temperatures below critical values.

Satellite tagging offers a means to investigate thermoregulatory behaviours by simultaneously recording depth and ambient temperature. However, transmissions via satellite are typically restricted to summarised forms. The detailed patterns of vertical movement necessary to reconstruct relationships between temperature and depth generally require physical recovery of the tag, in order to access the high-frequency data stored in the tag’s archive. Although this is a rare event^9,10^, due to the unpredictability of movements post-tagging and the open ocean environments inhabited by pelagic sharks, such tags are occasionally recovered and provide access to data that is sampled at sufficient temporal scales to investigate hypotheses concerning the drivers of movement patterns^11^.

Oceanic whitetip sharks, *Carcharhinus longimanus*, (OWTs) are large, epipelagic, predatory ectotherms (maximum body mass >150 kg^12^) that are globally distributed in warm-temperate and tropical oceans^13^. Here, we analyse high-resolution data archived from 16 recovered tags deployed on OWTs in the western North Atlantic Ocean for a mean of 183 ± 91 days. These data are used to test the hypothesis that, in a seasonally changing water column, OWTs will change their vertical movement patterns to avoid prolonged exposure to the highest SSTs. Given the shape of the typical thermal performance curve, we predict a distinct upper temperature breakpoint in vertical behaviour. Further, we characterise the behaviours that these sharks use to maintain body temperature, and discuss the likely drivers of individual variation in these behaviours.

## Results

### Track summary

**Figure 2:**
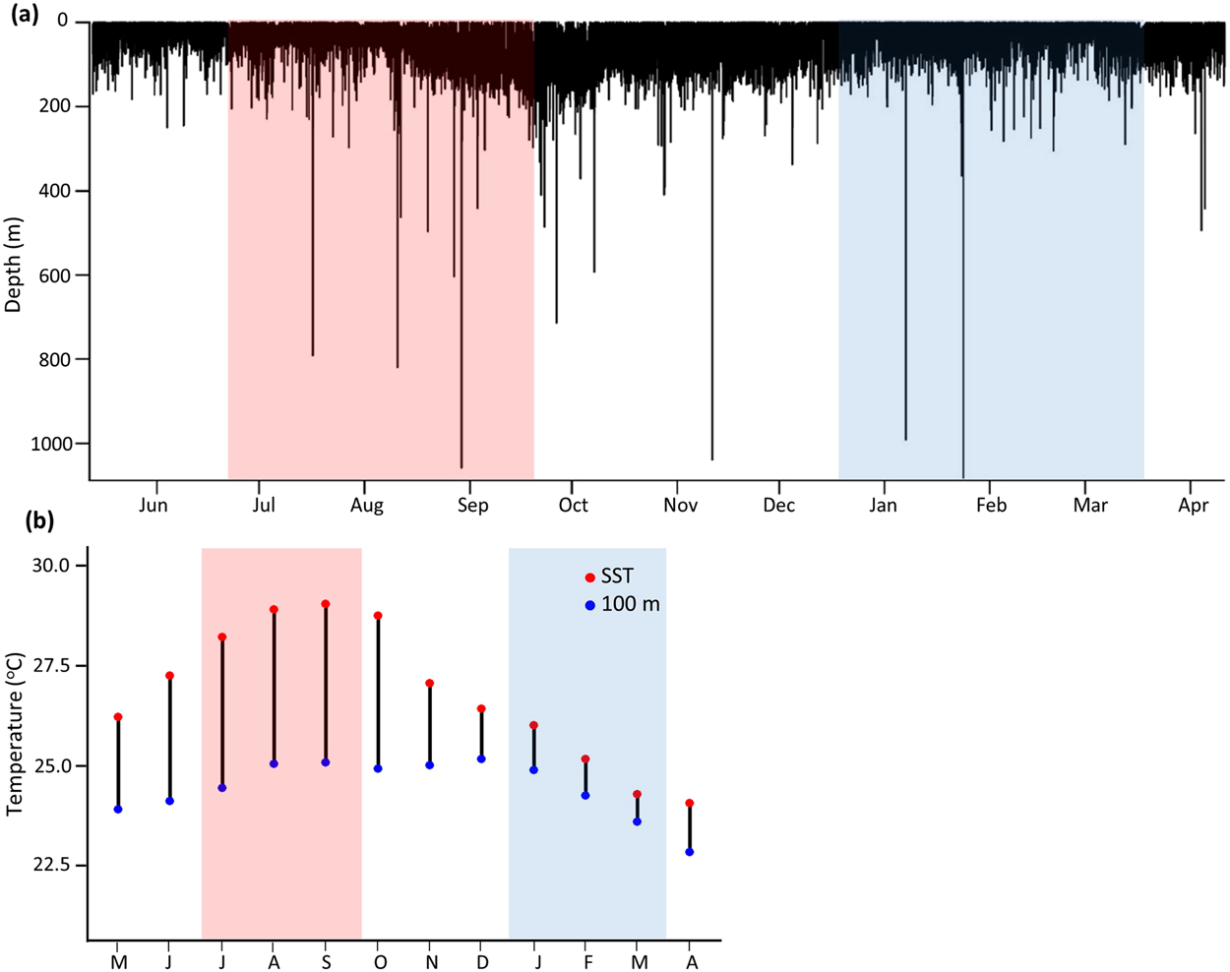
(**a**) Vertical movements of a tagged oceanic whitetips over 11 months. (**b**) The monthly mean temperature range between the surface and 100 meters depth for tagged oceanic whitetips. The red and blue shaded bars indicate timing of northern hemisphere summer and winter respectively.

Sixteen X-Tags were recovered from mature sharks providing access to 183 ± 91 days (mean ± standard deviation) of tracking data with a range of 20–333 days (see Supplementary Table S1 for more detailed deployment information). Individuals spent 99.64 ± 1.15% time in the upper 200 m of the water column, with a principal part of this time in the upper 100 m (90.10 ± 13.08%) (Fig. 2a). Mesopelagic Excursions (>200 m depths) occurred throughout the year (see Howey *et al*.^11^ for detailed analyses of these movements). Over the year the mixed layer depth (MLD) increased from a depth of 28.07 ± 10.11 m in summer (21^st^ June – 22^nd^ September) to 64.85 ± 24.93 m in winter (21^st^ December – 20^th^ March) as the water column became increasingly well-mixed. Daily mean sea surface temperature (SST) decreased from 28.55 ± 0.81 ^o^C during the summer months to 25.59 ± 1.07 ^o^C during winter months. The mean temperature range from the sea surface to 100 m depth also decreased from 3.88 ± 0.72 ^o^C in summer to 0.99 ± 0.39 ^o^C in winter (Fig. 2b).

### Oscillatory diving behaviours and environmental conditions

**Figure 3:**
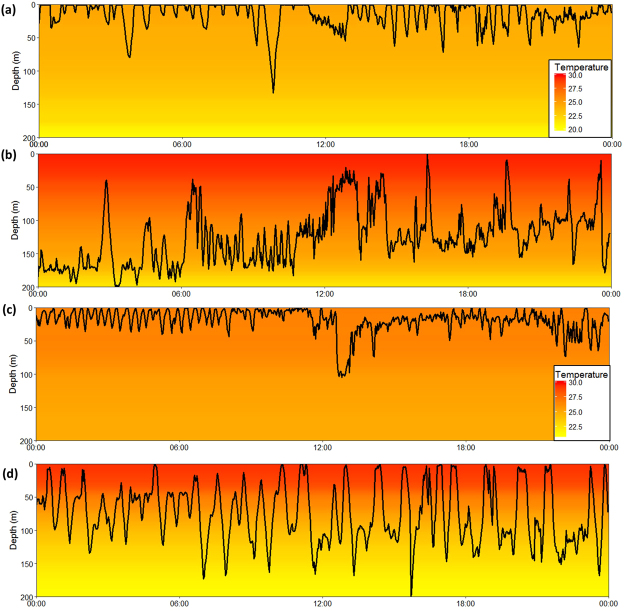
Daily oscillatory behaviour in a (**a**) winter and (**b**) summer water column for OWT8, and (**c**) winter and (**d**) summer for OWT7. Temperatures are averaged for the months of February and September respectively for 10 m depth bins. Depth trace is a 24 hour track sampled at a two minute frequency. Note that temperature scale-bars are not consistent between individual sharks.

Tagged OWTs continually descended and ascended through the water column. Continuous wavelet transformation analysis was used to estimate daily mean cycle length (time to complete an oscillation) and daily mean amplitude (depth of oscillation) (see Fig. 1b) of these vertical movements. Vertical distribution and cyclic characteristics changed with environmental conditions (Figs 1b, 3). An initial visual inspection of the depth profile indicated that most time was spent in the top 50 m of the water column, especially in winter months when it was cooler and vertical mixing was greater (Fig. 3a,c). As the SST increased in summer months, depth profiles displayed avoidance of the surface 50 m where waters were warmest (Fig. 3b) or larger and longer cycle oscillations (Fig. 3d).

**Figure 4:**
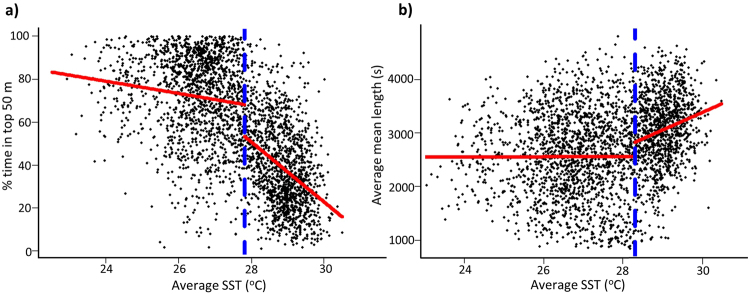
Relationship between SST and (**a**) daily % time spent in the top 50 m and (**b**) daily mean cycle length (s). Red and blue lines display results of piecewise regression with the blue line indicating the breakpoint, and the red lines the linear relationship either side of this breakpoint.

Three generalised linear mixed models were built to investigate relationships among daily means of environmental parameters, vertical distribution and oscillatory behaviour. All three models retained SST and MLD as explanatory variables and removed animal length in the model selection process (Table 1). A total of 35% of the variation in mean daily depth was explained by our first model with 21% attributed to variation in SST. Mean daily depth increased with SST, attaining a mean of 33.68 ± 15.83 m in winter and 56.50 ± 20.02 m in summer. Our second model explained 47% of the variation in the daily proportion of time spent in the top 50 m, with SST accounting for 31% of this variation (Table 1). The proportion of time spent in the top 50 m displayed a negative relationship with SST (Fig. 4a). Although model selection retained SST and MLD as explanatory variables of mean cycle length, these parameters did not explain a substantial proportion of the variation (Table 1). For all three models, conditional R^2^ values and individual OWT plots suggest high inter-individual variability in vertical movement behaviours (see Supplementary Figure S1).

**Table 1:** Subset of model comparisons using Akaike’s Information Criterion (AIC), Bayesian Information Criterion (BIC) and conditional (R^2^c) and marginal (R^2^m) R^2^ values.

Model	DF	AIC	BIC	ΔAIC	wAIC	R^2^c	R^2^m
**1. Mean depth** ~ **SST + MLD**	2839	21634	21682		0.645	0.35	0.23
Mean depth ~ SST + MLD + Length	2839	21636	21689	2	0.355	0.35	0.24
Mean depth ~ SST	2840	21671	21713	37	0.00	0.33	0.21
Mean depth ~ 1	2841	21693	21729	59	0.00	0.32	0
**2. Prop50 ~ SST + MLD**	2839	7104	7152		0.664	0.46	0.32
Prop50 ~ SST + MLD + Length	2839	7106	7159	2	0.336	0.46	0.33
Prop50~ SST	2840	7140	7182	36	0.00	0.46	0.31
Prop50~ 1	2841	7170	7206	47	0.00	0.62	0
**3. Mean cycle length ~ SST + MLD**	2839	43765	43812		0.668	0.43	0.05
Mean cycle length ~ SST + MLD + Length	2839	43767	43820	2	0.246	0.43	0.05
Mean cycle length ~ SST	2840	43814	43856	49	0.00	0.40	0.03
Mean cycle length ~ 1	2841	43818	43854	53	0.00	0.41	0

ΔAIC displays deviance in AIC scores from top-ranked models. All models are generalised linear mixed models and were ran using the nlme package in R with shark identity as a random variable. Proportion of time spent in the top 50 m (Prop50) was logit transformed prior to analysis. All null models include the random effect. To see all models involved in the model selection process, see Supplementary Table S2.

### Vertical behavioural breakpoints

A breakpoint of approximately 28 ^o^C occurred in the relationships of both daily proportion of time spent in the top 50 m and mean cycle length with SST (Fig. 4). Above 27.8 ^o^C the weak negative relationship between mean SST and proportion of time spent in the top 50 m steepened considerably from a slope of −2.86 to −13.99 (Fig. 4a). The mean proportion of time spent in the top 50 m of the water column at SSTs above and below 27.8 ^o^C was 38.21 ± 19.44% and 71.15 ± 20.24% respectively. Above this breakpoint, individuals rarely spent more than 80% of their time in the top 50 m. Below a 28.2 ^o^C breakpoint there was no apparent relationship between mean cycle length and SST (Fig. 4b). For days exceeding this breakpoint, a positive relationship with a slope of approximately 329 s was found. Mean cycle length increased from 2570 ± 793 s when SST was below 28.2 ^o^C to 3060 ± 677 s when SST was above 28.2 ^o^C. Mean values for other vertical movement parameters above and below an estimated 28 ^o^C breakpoint are listed in Table 2. Notably, mean SST increased by more than two degrees between these two groups, however the overall mean temperature experienced by tagged sharks increased by less than one degree. The inclusion of the breakpoint term minimised the AIC for both full models, however, the result was more variable when assessed on an individual basis (Tables S3, S4). The AIC suggested that in 11 of 13 individuals the inclusion of a breakpoint between 27–29 ^o^C had greater support than a single linear relationship (ΔAIC = 6–122) for the daily proportion of time spent in the top 50 m, and nine individuals the inclusion of the breakpoint for mean cycle length had greater support than the single linear relationship (ΔAIC = 6–60).

**Table 2:** Mean (±standard deviation) of daily vertical movement behaviours above and below 28 ^o^C.

Parameter	Below 28 ^o^C	Above 28 ^o^C
Mean SST	26.56 ± 0.96 ^o^C	28.91 ± 0.48 ^o^C
Mean temperature	25.70 ± 0.83 ^o^C	26.66 ± 0.79 ^o^C
Mean depth	38.63 ± 17.0 m	63.06 ± 18.20 m
% time spent in top 50 m	70.61 ± 20.41%	37.57 ± 19.17%
Mean cycle length of oscillations	2576 ± 788 s	3013 ± 711 s
Mean amplitude of oscillations	14.76 ± 6.25 m	20.74 ± 8.0 m
Mean MLD	39.20 ± 23.36 m	32.01 ± 10.65 m

### Cluster analysis

**Figure 5:**
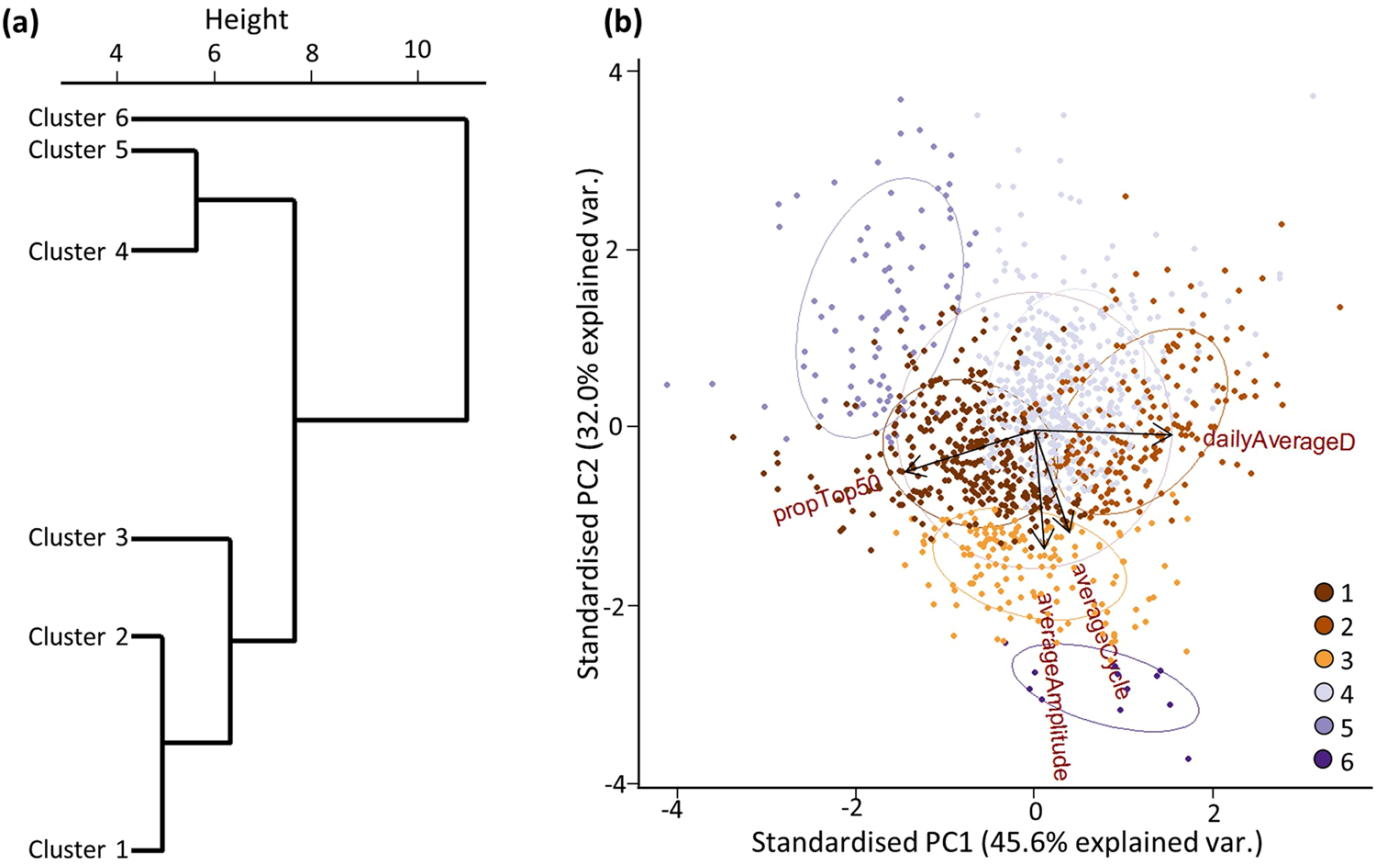
Results from multivariate analysis of daily data where SST >28 ^o^C. Input variables were daily values of proportion of time spent in the top 50 m, mean depth, mean cycle length and mean amplitude. (**a**) Dendrogram of vertical movement behaviour determined from hierarchical cluster analysis. (**b**) The two principal components scores are plotted for daily data. Points are coloured by their cluster group.

**Figure 6:**
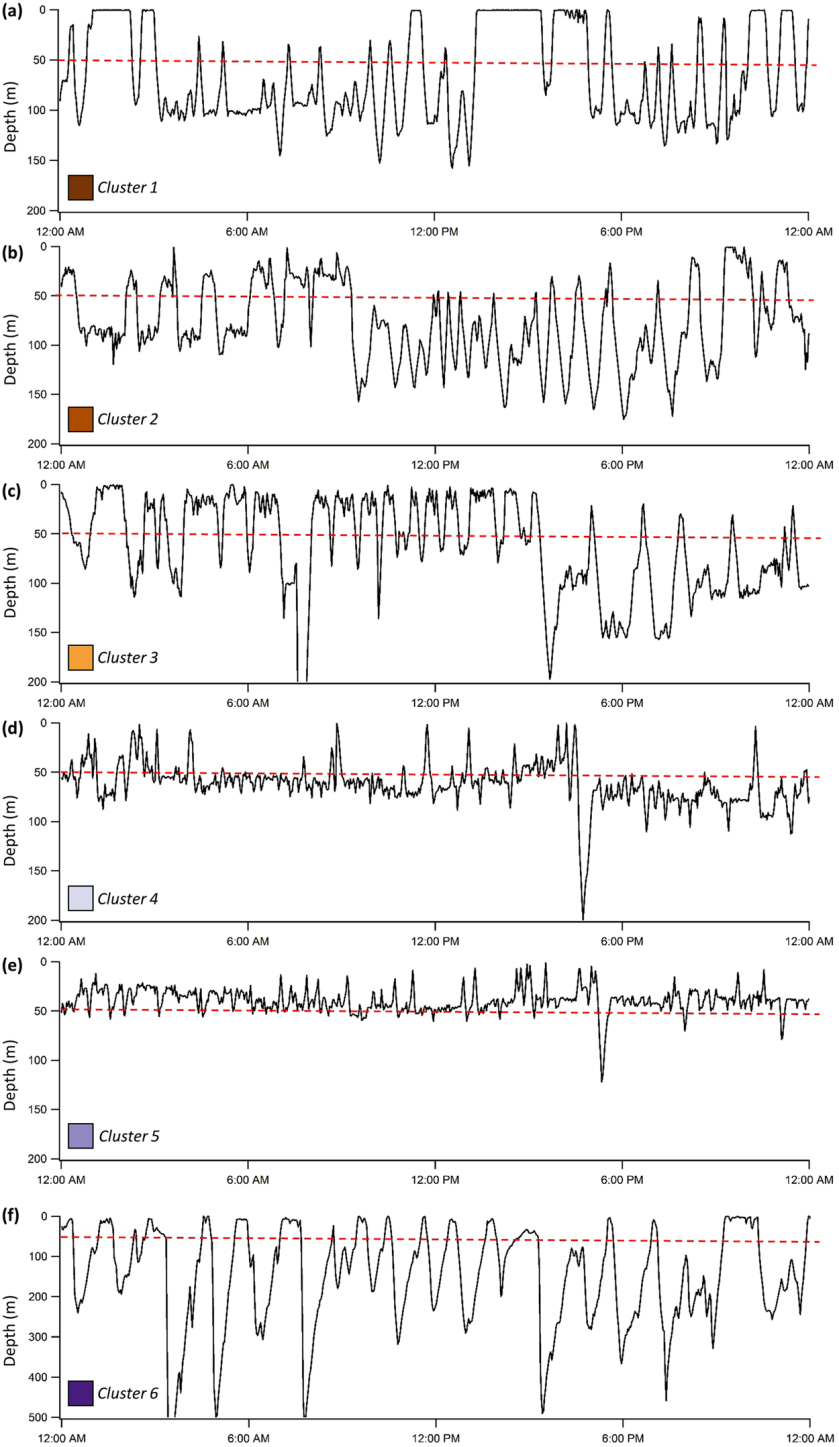
24 hour depth profiles representative of each group derived from the hierarchical cluster analysis. (**a**) Medium % top 50, medium cycles, low amplitude, (**b**) low % top 50, long cycles, low amplitude, (**c**) medium % top 50, long cycles, medium amplitude, (**d**) low % top 50, short cycles, low amplitude, (**e**) high % top 50, short cycles, low amplitude, (**f**) medium % top 50, long cycles, high amplitude. Note that the scale on the y-axis differs for Cluster 6.

To further classify vertical movements when surface waters warmed, metrics of vertical movement including mean depth, proportion of time spent in the top 50 m, mean cycle length and mean amplitude were used for a hierarchical cluster analysis for individual days when the SST exceeded 28 ^o^C. Three individual OWTs were excluded due to an insufficient sample size of days meeting this criteria (<50 days). Six clusters emerged from this analysis and the PCA indicated that 77.6% of the variation in these vertical movement behaviour could be explained by the first two components (Fig. 5). The proportion of time spent at depths <50 m and mean daily depth each contributed to more than 40% of the variation in PC1, and mean daily cycle length and mean daily amplitude each contributed to more than 35% of the variation in PC2. Characteristics of each cluster are summarised in Table 3, and a representative example of each are displayed in Fig. 6. Cluster 4 was the most common (37.21% of days) and was characterised by a low proportion of time spent in the top 50 m and short cycle (approximately 44 minutes) and low amplitude oscillations (Table 3). Cluster 1 was the second most common (27.83%) and showed medium levels of both time spent in the top 50 m and cycle length. Clusters 2 and 3 both showed long cycles of approximately one hour with cluster 2 being distinguished by very low use of the top 50 m of the water column. Cluster 5 was characterised by higher use of the top 50 m and oscillations with relatively small amplitude and short cycles. Although the proportion of the time spent within the top 50 m for sharks within this cluster was high, the mean MLD was relatively shallow and the mean time spent outside the ML was comparable with other clusters (Table 1). Cluster 6 only comprised 0.93% of the data, and was characterised by oscillations with the longest cycle and highest amplitude. Days associated with this cluster were found to have repeated deep descents to depths greater than 200 m (Fig. 6f). In summary, the clusters were composed of different combinations of vertical behaviours that collectively avoided prolonged exposure to the warmest temperatures found in the surface layers. In addition, with the exception of cluster 1, the mean daily temperature was maintained within a standard deviation of the overall mean daily temperature recorded by the tags (26.14 ± 0.94 ^o^C).

**Table 3:** Summarised characteristics of each cluster derived from the hierarchical cluster analysis.

Cluster ID	1	2	3	4	5	6
*% days*	27.83%	15.12%	12.17%	37.21%	6.74%	0.93%
*Mean % time top 50*	51.72 ± 12.44%	17.18 ± 7.69%	45.13 ± 13.17%	25.98 ± 9.07%	67.78 ± 14.22%	40.68 ± 10.64%
*Mean depth*	50.42 ± 11.99 m	82.43 ± 13.04 m	64.41 ± 17.85 m	68.90 ± 10.60 m	38.41 ± 10.95 m	95.32 ± 25.22 m
*Mean cycle length*	3168 ± 509 s	3651 ± 390 s	3620 ± 410 s	2637 ± 528 s	1914 ± 642 s	3995 ± 357 s
*Mean amplitude*	19.82 ± 4.72 m	17.51 ± 4.59 m	33.02 ± 6.81 m	19.01 ± 4.81 m	14.43 ± 3.64 m	60.91 ± 8.50 m
*Mean temperature*	27.13 ± 0.64 ^o^C	26.53 ± 0.65 ^o^C	26.57 ± 0.82 ^o^C	26.41 ± 0.70 ^o^C	26.72 ± 1.11 ^o^C	25.73 ± 0.81 ^o^C
*Mean MLD*	31.77 ± 11.37 m	39.68 ± 10.28 m	30.39 ± 9.62 m	32.15 ± 8.55 m	20.84 ± 7.11 m	33.88 ± 8.34 m
*Mean % time outside ML*	63.48 ± 15.81%	86.98 ± 7.22%	64.30 ± 14.44%	84.66 ± 8.81%	67.54 ± 22.44%	63.26 ± 10.36%
*Mean SST*	28.84 ± 0.49 ^o^C	29.21 ± 0.43 ^o^C	28.91 ± 0.42 ^o^C	28.93 ± 0.44 ^o^C	28.58 ± 0.46 ^o^C	29.28 ± 0.22 ^o^C
*Behaviour*	Medium % top 50, medium cycles, low amplitude	Low % top 50, long cycles, low amplitude	Medium % top 50, long cycles, medium amplitude	Low % top 50, short cycles, low amplitude	High % top 50, short cycles, low amplitude	Medium % top 50, long cycles, high amplitude

Values are mean ± standard deviation.

### Inter-individual variability

**Figure 7:**
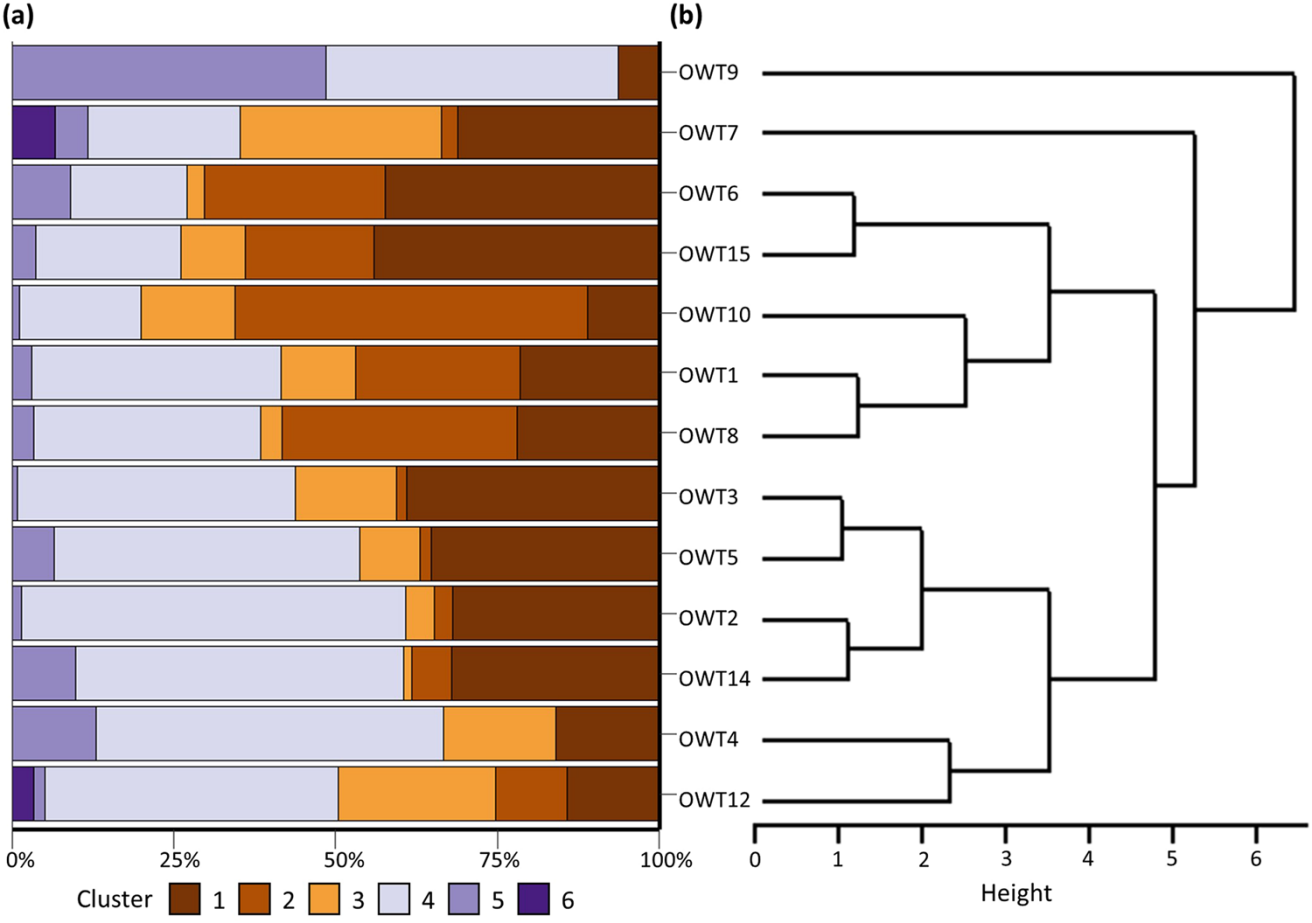
The percentage of time each individual OWT spent in each calculated cluster. X-axis ordered based on similarity of cluster composition between OWTs. OWT11, OWT13 and OWT16 were excluded from this analysis due to insufficient sample size in days with SST >28 ^o^C.

The clusters were further examined to investigate which individuals were most similarly grouped. The proportion of time spent in each cluster varied among individuals, and an additional cluster analysis that focused on this parameter revealed distinct groupings (Fig. 7a,b). Some clear patterns emerged from this analysis. Firstly, the only male shark tagged by the study (OWT9) displayed a pattern of vertical movement that was distinct from all other tagged sharks. Secondly, the majority of the female sharks were pooled into two clusters. The first (OWT2, OWT3, OWT4, OWT5, OWT12 and OWT14) was characterised by individuals that spent very little time in surface waters and underwent shorter cycle oscillations (Fig. 7a). The second group (OWT1, OWT6, OWT8, OWT10 and OWT15) tended to avoid surface waters and underwent long cycle oscillations.

## Discussion

Theories of foraging ecology and thermal physiology suggest that large animals found in tropical environments should maintain an optimal and stable body temperature range^4,14,15^. This stability is required because a number of physiological processes, such as growth, have a tendency to increase with temperature. Thus, an individual will have optimal performance if temperatures are maintained within the upper limits of this range, assuming there is sufficient prey available to support the relatively high metabolic rates that this entails^15–17^. Due to the asymmetry of the thermal performance curve, slight increases in temperature above optimum can result in rapid declines in performance^5^. This will require aquatic ectotherms to vacate environments when temperatures become unfavourable in order to maintain performance. We predicted that OWTs would modify their diving behaviour as the thermal structure of the water column changed seasonally and that sharp behavioural breakpoints would be evident at higher temperatures that might indicate the upper limits of thermal performance curves. In cooler months, when SST was lower than 28 ^o^C, OWTs spent a high proportion of their time in the top 50 m of the water column. Beyond this limit, OWTs avoided the top 50 m where waters were the warmest, and/or performed longer cycle and higher amplitude oscillations from the surface to deeper, cooler waters. As a result the mean daily temperature experienced by the shark shifted by only a degree (25.70 ± 0.83 ^o^C below 28 ^o^C SST, 26.66 ± 0.79 ^o^C above 28 ^o^C), despite much larger changes in SST in the top 50 m of the water column across the season.

Evidence for a behavioural breakpoint with changes in temperature are consistent with the hypothesis that OWT sharks have behavioural strategies for thermoregulation. Our data suggests that OWT sharks in the western North Atlantic Ocean face metabolic challenges similar to some terrestrial ectotherms^6^ in having to cope with habitats close to upper thermal limits and potential overheating. To date, most studies of strategies for thermoregulation in marine ectotherms have focused on behaviours that might function to regain heat lost after descents into deeper and cooler waters to feed on the deep scattering layer^18,19^. There is, however, evidence that endothermic fishes that inhabit cooler waters might face similar physiological challenges when migrating through warmer latitudes. For example, Teo *et al*.^20^ hypothesised that Atlantic bluefin tuna, *Thunnus thynnus*, descended to deeper waters on entry and exit to the Gulf of Mexico in order to avoid warm surface waters. Similarly, female porbeagle sharks, *Lamna nasus*, are thought to avoid warm surface waters (22–29 ^o^C) in the Gulf Stream and Sargasso Sea, with no individual recorded in waters with a six-hour mean greater than 21.9 ^o^C^21^. In the northern Pacific, salmon sharks, *L. ditropis*, have been found to descend to deeper, cooler waters as they migrate south towards lower latitudes^22,23^. Tagged individuals of this species generally positioned themselves at deeper and cooler depths when surface temperatures exceeded 23 ^o^C in a multi-year satellite tag dataset^23^. These observations suggest that breakpoints in vertical movement behaviours at high SSTs might also exist in other species, both endothermic and ectothermic.

Although OWTs displayed a variety of vertical movement behaviours that we suggest avoided prolonged exposure to surface waters when SST warmed above 28 ^o^C, these behaviours were not necessarily consistent among tagged individuals. Such inter-individual variation has been reported by many studies tracking both the horizontal and vertical movements of sharks^24–26^. In our study the most important differences in vertical behaviours among OWT sharks occurred in the proportion of time individuals resided in the top 50 m of the water column and in the length and amplitude of their vertical oscillations, although it is important to note that despite this variation in strategies, mean water temperatures experienced by the sharks were always maintained between 25–27 ^o^C. Whereas at present we cannot be certain of the drivers of these individual variations in movement patterns, differences could reflect the influence of prey availability, ontogenetic and reproductive stage, intraspecific interactions and interaction with predators, and/or differences in thermal physiology.

Regardless of the use of horizontal space by an individual shark, all OWTs underwent mesopelagic excursions to depths of up to 1190 m, a behaviour that may represent prey searching^11^. Around the tagging locality of Cat Island, OWTs are thought to feed predominately on large pelagic teleosts and after movement away from this region, their diet alters to include a significantly higher proportion of squid^27^. Such changes in diet and the consequent distribution of potential prey may drive differences in vertical behaviours among individuals, by determining whether or not an individual needs to access warmer surface waters. If prey are available at depth, an OWT will not need to enter the top 50 m, whereas if prey are only available in surface waters, an individual may undergo longer oscillations to cooler depths in order to dissipate excess heat gained while foraging in surface layers. Similarly, individual dietary specialisation may drive differences in movements if preferred dietary items differ in their vertical distributions. Comparing the isotopic composition of tagged individuals may help to assess differences in vertical behaviours.

We tagged sharks at an aggregation site off Cat Island, in the Bahamas. The sex ratio of the aggregation is dominated by females that either remain in the region year round or undergo long-distance migrations that commence around July^28^, coinciding with seasonal warming of surface waters. The onset of changes in vertical habitat use we observed – higher-amplitude and longer cycle oscillations and avoidance of waters >28 °C – were not confined to those individuals that migrated, since one female shark (OWT3) that remained near the Bahamas also exhibited the same pattern of avoidance of the upper 50 m depths as surface waters warmed. In contrast, the only male tagged by our study remained near the Bahamas (except for a three-week period from late August through early September), but did not have any obvious strategy in vertical movements that avoided the warmest surface waters. While bearing in mind the limitations of generalisations based on the results from a single tag, it might be possible that this difference between males and females occurs because females have a greater need to maintain a narrower thermal range due to higher energetic demands^1^. A larger number of males need to be sampled to investigate this possibility.

The total length of the tagged individuals did not appear to drive any of the variation in movement patterns among individuals and, as a majority were mature females, we could not explore the effect of sex or life history stage in any great detail. As OWTs are thought to have biennial reproductive cycles^29^, it is possible that differences in patterns of vertical movement we documented among females may arise from reproductive status, as developing pups may make greater metabolic demands and thus require higher rates of food intake and/or maintenance of body temperatures within a smaller temperature range.

Our results were consistent with the hypothesis that OWT sharks display behavioural strategies that maintain stable body temperatures in a warm and oligotrophic tropical environment. An understanding of relationships between vertical movement behaviours and ambient temperature is essential if we are to document the capacity for ectothermic sharks to buffer the impact of changing environmental conditions, particularly warming oceans^6^. Irrespective of SST, sharks experienced very similar mean daily temperatures throughout the time they were tagged. This was achieved in a variety of ways when waters warmed above 28 ^o^C. At temperatures below this breakpoint, OWTs continually displayed oscillatory diving behaviour, even when the water column was well-mixed down to 100 m, suggesting that thermoregulation was not the primary driver of these movements (Fig. 3a,c). We also acknowledge that some other unmeasured environmental variable, such as light or water quality, prompted the observed changes in vertical movement behaviours. Nevertheless if this was the case, OWTs would likely continue to benefit from the maintenance of a stable body temperature.

Although these behaviours demonstrate the potential of OWTs to adapt to a warming ocean, there are several implications of these results. Given variability in species thermal optima, potential habitat mismatches between OWT and their prey are possible in the future if warming increases thermal habitat close to upper thermal limits, reducing the zone of overlap in which OWTs can feed^30^. This avoidance of surface waters will reduce the vulnerability of these sharks to fishing gears targeting this zone, but may increase their vulnerability to deeper-set longlines by compressing the zone of available habitat for OWTs^31^, magnifying the spatial overlap of OWT distributions with pelagic longline fisheries that already occurs on a horizontal scale^32^. In winter months, when the top 100 m becomes well-mixed, these problems may be compounded if winter SSTs begin to attain upper thermal limits and sharks have to descend to greater depths to reach cooler temperatures. Fortunately, in recent years, SSTs in regions around the Bahamas have followed a cooling trend in winter months^33^, however, this may be a problem confronting the species in other regions. Additionally, other impacts of climate change may also influence the response of OWTs to a warming ocean. Lower oxygen content of warming waters may reduce the ability of sharks to chase prey^34^, escalating the difficulties facing OWTs in an oligotrophic pelagic environment. Several studies have reported declines in the abundance and size of the OWT^35–37^. Although the retention of OWTs by fisheries has generally been banned, high spatial overlap with targeted species means capture rates are unlikely to decline and other strategies are needed to conserve populations^38^. Anticipating future seasonal and overall shifts in vertical distribution will have important implications for developing and implementing effective mitigation measures such as spatial and/or temporal closures.

In order to measure the impacts of warming on the physiological performance of OWTs, we need to quantify the upper limits of the thermal performance curve for the species. Traditionally, respirometry approaches have provided the data on metabolic rate and cost of transport of fishes under different temperature regimes^16^, although this usually requires captive trials in laboratories and are unlikely to be a logistically feasible option. However, accelerometry now offers a viable technique for calculating the metabolic rate of these animals^39^ and deployment of tags containing accelerometers in different seasons to compare energy expenditure at SSTs above and below 28 ^o^C will be crucial for predicting the future consequences of a warming ocean. It is also vital that we continue to explore how temperature structures the vertical movements of sharks and incorporate our findings into projections of future distribution shifts of these animals. As the effect of temperature may vary between regions for a given species^1^, it will also be useful to assess vertical movements on a broader geographical range.

## Materials and Methods

### Data collection

Oceanic whitetip sharks were captured and tagged with Standard Rate (SR) X-Tags (Microwave Telemetry, Inc., Columbia, MD) near Cat Island, The Bahamas (24.12°N, 75.28°W) during May 2011–2013. All equipment, tagging procedures and analysis of horizontal data are described in detail elsewhere^11,28^. In brief, a handheld tagging applicator was used to insert X-Tags into the dorsal musculature of captured sharks. These tags recorded depth (0.34 m resolution), temperature (0.16–0.23 ^o^C resolution), and light level at two-minute intervals. Physical recovery of the tags allowed for whole archival datasets to be downloaded. This sampling frequency was assessed to adequately capture the vertical movements of these sharks by comparing profiles obtained at one-second intervals with a two-minute subsample recorded by an individual tagged with a PD3GT data logger (1 Hz, ± 0.25 m resolution) (Little Leonardo, Tokyo, Japan)^11,40^. Prior to analysis, the first and last day of the tag deployment was discarded from each dataset.

Research was conducted under the Cape Eleuthera Institute (CEI) research permit (MAF/FIS/17 & MAF/FIS/34) issued by the Bahamian Department of Marine Resources in accordance with CEI animal care protocols developed within the guidelines of the Association for the Study of Animal Behaviour and the Animal Behavior Society.

### Data analysis

We used R 3.4.0 for all data analysis^41^, except where stated otherwise.

#### Environmental parameters

Daily mean sea surface temperatures (SST) and mixed layer depths (MLD) were calculated using depth and temperature data collected by tagged individuals. SST was calculated as the mean temperature in the uppermost 5 m of the water column each day. MLD was calculated using the threshold method^42^. We chose this approach as the tags did not record salinity, preventing us from using density criteria, and the sampling interval prevented the sharp gradient-resolved profiles required for gradient criterion^42^. The threshold method involves determining the depth at which there was a change in temperature from a near-surface reference depth by more than a chosen threshold value. The daily mean temperature from the 7.5–12.5 m depth bin was used as a reference value to avoid the diurnal variation in the surface layer^42^ and a classical threshold value of 0.5 ^o^C^43^ was selected based on visual inspection of daily temperature-depth profiles recorded by the tags.

#### Vertical movement behaviours

The cyclic nature and amplitude of vertical movements were analysed using the software Igor Pro (WaveMetrics Inc., Lake Oswego, OR, USA) with the package Ethographer^44^. Continuous wavelet transformation was applied to the entire depth dataset of each shark. Based on visual inspection of the depth profiles, minimum and maximum cycle length in Ethographer were set to 10 minutes and 90 minutes, respectively. The mean cycle length (seconds) and amplitude (meters) (see Fig. 1b) of these cycles were extracted and a daily mean was calculated.

For each day, mean depth and temperature, the mean time spent outside of the mixed layer and the proportion of time spent within distinct depth ranges were also calculated. Preliminary analysis revealed that the proportion of time spent in the top 50 m displayed the strongest relationship with environmental parameters. For this reason this depth range was used for further analyses.

#### Generalised linear mixed models

We built three generalised linear mixed models (GLMMs) using the nlme^45^ package in R to investigate relationships among daily means of environmental parameters, vertical distribution and oscillatory behaviour. The explanatory variables for each model were initially set as SST, MLD and total length of the shark (hereafter length), and shark identity as a random variable. Mean depth, proportion of time in the upper 50 m and mean cycle length were all sequentially modelled as response variables. Proportion of time spent in the top 50 m was logit transformed prior to analysis. We used the corAR1 function to account for temporal auto-correlation in our datasets^46^. All possible combinations of the explanatory variables were modelled, and resulting models were compared and ranked using weights of Akaike’s information criterion (AIC).

#### Breakpoint analysis

Potential breakpoints in the relationships between SST and vertical movement behaviours were initially identified through visual inspection. Breakpoints occur where there is a sudden and sharp change in the directionality of a linear relationship. Piecewise regression was used to statistically estimate these breakpoints by fitting separate slopes for data above and below modelled breakpoints until the best fit was found. This method has traditionally been used to successfully estimate breakpoint positions and ecological thresholds in species-habitat relationships in the terrestrial realm^47^, and has more recently been applied in predicting optimal temperatures for the activity of ectothermic fishes such as Arctic char, *Salvelinus alpinus*^48^. We used an iterative searching method with SST set as the explanatory variable, and mean cycle length and proportion of time spent in the upper 50 m each set in turn as the dependent variable. Each unique SST was used as a breakpoint and a linear regression was run for all possible breakpoints. The model with the lowest residual mean squared error (MSE) was used to select the breakpoint. The AIC of models with and without the breakpoint term were compared for the full dataset as well as for each individual shark.

#### Multivariate analysis – hierarchical clustering

Where distinct breakpoints in vertical movement behaviours in response to SST were found, we applied a hierarchical cluster analysis to categorise vertical behaviours employed in the upper thermal range. This method has been used by earlier studies to successfully distinguish among behavioural modes of vertical movement in marine megafauna^9,18^. The proportion of time spent in the top 50 m, mean depth, mean cycle length and mean amplitude were summarised to daily values and scaled using the scale function in R. Cluster analysis was applied on these variables using the hclust function in R based on a dissimilarity matrix produced from Euclidean distances and complete linkage as the agglomeration method. We then used principal components analysis (PCA) on these clustered data to determine if vertical movement behaviours differed among the resulting groups. Vertical movement behaviours were then examined by clusters and were subjectively broken into groups based on the distance between clusters and the cluster characteristics. Sharks with less than 50 days of data within the given SST range were not included in this analysis. To determine which individuals were most similarly grouped, the same clustering procedure was used on the proportion of time spent in each cluster.

### Data availability

On acceptance of manuscript we intend to deposit data to the KNB Data Repository.

## Electronic supplementary material

Supplementary information
