# Supplementary material for: Temperature and the vertical movements of oceanic whitetip sharks, Carcharhinus longimanus
Source: Sci Rep. 2018 May 29;8:8351. doi: 10.1038/s41598-018-26485-3 (PMC5974137; doi:10.1038/s41598-018-26485-3)
Supplement: Supplementary file 1 — Supplementary information [file 41598_2018_26485_MOESM1_ESM.pdf]

Supplementary Information

Temperature and the vertical movements of oceanic whitetip sharks, *Carcharhinus longimanus*

Samantha Andrzejaczek[1,2*], Adrian C. Gleiss[3], Lance K. B. Jordan[4], Charitha B. Pattiaratchi[1], Lucy A. Howey[4], Edward J. Brooks[5] and Mark G. Meekan[2]

[1]School of Civil, Environmental and Mining Engineering & The UWA Oceans Institute, The University of Western Australia, Crawley, WA, 6009, Australia

[2]The Australian Institute of Marine Science, Crawley WA, 6009, Australia

[3]Centre for Fish and Fisheries Research, Murdoch University, Murdoch, WA, 6150, Australia

[4]Microwave Telemetry, Inc., Columbia, Maryland, 21045, USA

[5]Cape Eleuthera Institute, The Bahamas

*****sandrzejaczek@gmail.com

Table S1. Summary of tagging information and biological information of the tagged individuals.

Shark ID	Deployment date	Days at liberty	Fork length (cm)	Sex	Pop-off latitude (°N)	Pop-off longitude (°S)	Mean (± SD) depth (m)	Mean (± SD) temperature (°C)	Maximum depth (m)	Minimum temperature (°C)
OWT1	5/1/2011	184	218	F	22.89	74.68	59.92 ± 45.02	26.35 ± 1.68	1081.9	7.75
OWT2	5/4/2011	153	189	F	23.20	74.91	46.02 ± 38.52	26.16 ± 1.87	1008.7	8.54
OWT3	5/4/2011	245	214	F	23.73	74.93	50.03 ± 37.29	26.59 ± 1.46	751.8	10.89
OWT4	5/1/2011	153	233	F	24.36	75.37	41.66 ± 37.42	25.98 ± 1.66	720.2	7.90
OWT5	5/7/2012	245	212	F	24.60	75.50	43.32 ± 33.9	26.18 ± 1.56	577.3	13.53
OWT6	5/7/2012	150	179	F	22.69	73.38	50.8 ± 39.9	27.09 ± 1.65	635.1	11.04
OWT7	5/11/2012	278	228	F	23.94	74.93	45.37 ± 48.83	26.34 ± 1.74	1190.2	7.43
OWT8	5/12/2012	335	212	F	24.75	75.52	45.82 ± 40.55	25.29 ± 1.54	1081.9	6.79
OWT9	5/12/2012	325	195	M	24.01	74.46	57.65 ± 41.11	25.33 ± 1.56	875.2	9.48
OWT10	5/9/2012	112	216	F	24.22	75.32	74.94 ± 44.19	26.58 ± 1.70	845.6	9.32
OWT11	5/10/2012	60	168	F	24.67	76.05	37.28 ± 29.26	26.04 ± 1.26	420.6	17.63
OWT12	5/9/2012	245	210	F	21.77	74.20	48.64 ± 43.02	26.31 ± 1.65	895.3	9.48
OWT13	5/8/2013	83	198	M	23.11	75.02	42.04 ± 41.29	25.95 ± 1.32	855.3	9.63
OWT14	5/9/2013	174	183	F	25.30	76.34	46.63 ± 33.04	26.36 ± 1.56	442.1	17.94
OWT15	5/12/2013	202	203	F	24.63	75.83	45.04 ± 35.54	26.56 ± 1.53	838.5	9.32
OWT16	5/8/2013	22	203	F	24.83	75.70	39.42 ± 30.49	25.33 ± 0.94	232.0	20.21

Table S2. All models involved in the model selection process. Model comparisons were made using Akaike's Information Criterion (AIC), Bayesian Information Criterion (BIC) and conditional R^2 and marginal R^2 values. ΔAIC displays deviance in AIC scores from top ranked models. All models are generalised linear mixed models and were ran using the nlme package in R with shark identity as a random variable. Proportion of time spent in the top 50 m (Prop50) was logit transformed prior to analysis. All null models include the random effect.

Model	DF	AIC	BIC	ΔAIC	wAIC	R^2c	R^2m
Mean depth ~ SST + MLD + Length	2839	21636	21689	2	0.355	0.35	0.24
**Mean depth ~ SST + MLD**	**2839**	**21634**	**21682**		**0.645**	**0.35**	**0.23**
Mean depth ~ SST + Length	2840	21672	21720	38	0.00	0.33	0.22
Mean depth ~ MLD + Length	2840	21734	21781	100	0.00	0.06	0.006
Mean depth ~ MLD	2840	21659	21701	25	0.00	0.35	0.01
Mean depth ~ Length	2841	21694	21736	60	0.00	0.32	0.005
Mean depth ~ SST	2840	21671	21713	37	0.00	0.33	0.21
Mean depth ~ 1	2841	21693	21729	59	0.00	0.32	0
Model	DF	AIC	BIC	ΔAIC	wAIC	R^2c	R^2m
Prop50 ~ SST + MLD + Length	2839	7106	7159	2	0.336	0.46	0.33
Prop50 **~ SST + MLD**	**2839**	7104	7152		0.664	0.46	0.32
Prop50 ~ SST + Length	2840	7142	7189	38	0.00	0.45	0.31
Prop50 ~ MLD + Length	2840	7135	7183	31	0.00	0.65	0.01
Prop50 ~ MLD	2840	7134	7176	30	0.00	0.67	0.007
Prop50 h ~ Length	2841	7168	7210	64	0.00	0.59	0.005
Prop50 ~ SST	2840	7140	7182	36	0.00	0.31	0.46
Prop50 h ~ 1	2841	7167	7203	63	0.00	0.62	0
Model	DF	AIC	BIC	ΔAIC	wAIC	R^2c	R^2m
Mean cycle length ~ SST + MLD + Length	2839	43767	43820	2	0.246	0.43	0.05
**Mean cycle length ~ SST + MLD**	**2839**	**43765**	**43812**		**0.668**	**0.43**	**0.05**
Mean cycle length ~ SST + Length	2840	43815	43863	50	0.00	0.40	0.03
Mean cycle length ~ MLD + Length	2840	43771	43819	6	0.023	0.45	0.02
Mean cycle length ~ MLD	2840	43769	43811	4	0.063	0.34	0.009
Mean cycle length ~ Length	2841	43887	43917	122	0.00	0.41	0.005
Mean cycle length ~ SST	2840	43814	43856	49	0.00	0.40	0.03
Mean cycle length h ~ 1	2841	43818	43854	53	0.00	0.41	0

Table S3. Results from proportion time top 50 m ~ mean SST breakpoint analysis. Breakpoints were assessed for the full dataset and each individual shark. Model comparisons were made using Akaike's Information Criterion (AIC) between models with and without the breakpoint term. ΔAIC displays deviance in AIC scores between these two models. *Calculated breakpoint not robust. **Breakpoint term significantly improves model.

OWT ID	Estimated breakpoint	AIC breakpoint	AIC no breakpoint (ΔAIC)
All	27.8	25793	26111 (318)**
OWT1	27.2	1584	1611 (27)**
OWT2	28.4	1279	1306 (27)**
OWT3	27.4	1896	1940 (44)**
OWT4	28.1	1267	1353 (86)**
OWT5	27.6	2084	2163 (115) **
OWT6	28.9	1219	1253 (34) **
OWT7*	26.1	2390	2430 (40)
OWT8	27.5	2805	2927 (122) **
OWT9*	25.2	2812	2849 (37)
OWT10	28.2	939	945 (6) **
OWT12	28.3	1987	2029 (42) **
OWT14	28.6	1370	1379 (9) **
OWT15	27.5	1631	1683 (52) **

Table S4. Results from mean cycle length ~ mean SST breakpoint analysis. Breakpoints were assessed for the full dataset and each individual shark. Model comparisons were made using Akaike's Information Criterion (AIC) between models with and without the breakpoint term. ΔAIC displays deviance in AIC scores between these two models. *Calculated breakpoint not robust. **Breakpoint term significantly improves model.

OWT ID	Estimated breakpoint	AIC breakpoint	AIC no breakpoint (ΔAIC)
All	28.2	47351	47457 (106)**
OWT1	28.6	2826	2826 (0)
OWT2	28.4	2416	2428 (12)**
OWT3	27.4	3793	3806 (13)**
OWT4	27.5	2402	2430 (28)**
OWT5*	25.6	3735	3757 (22)
OWT6*	29.3	2290	2306 (16)
OWT7	28	4271	4295 (24)**
OWT8	27	5256	5271 (15)**
OWT9	27.4	5180	5240 (60)**
OWT10	28.4	1724	1730 (6)**
OWT12	27.4	3731	3744 (13)**
OWT14*	29.3	2648	2653 (5)
OWT15*	26.5	3099	3100 (1)

Figure S1. The relationship between daily averaged vertical movement behaviours and SST for each individual OWT. (a) Mean daily depth; (b) percentage time in the top 50 m; (c) mean cycle length (seconds); and (d) mean amplitude.

(a)

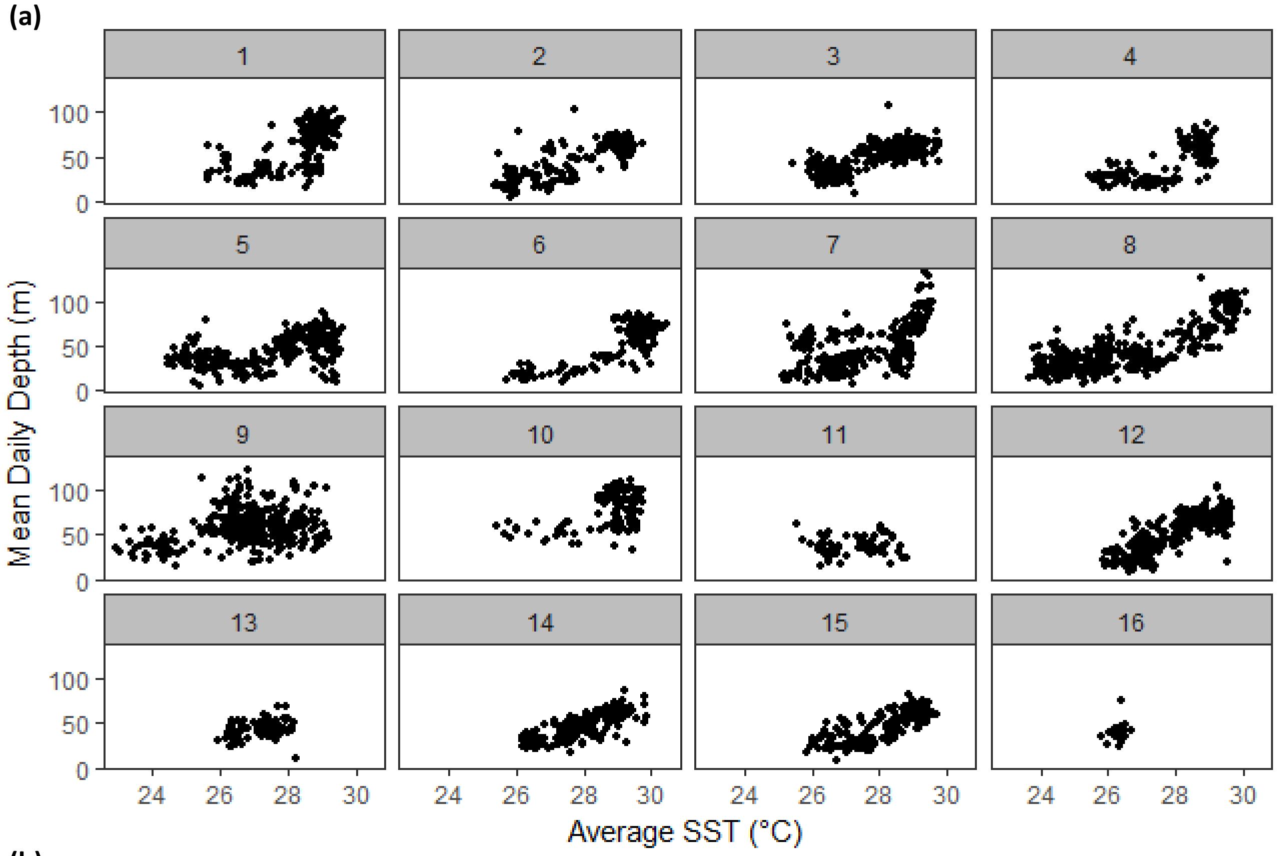

(b)

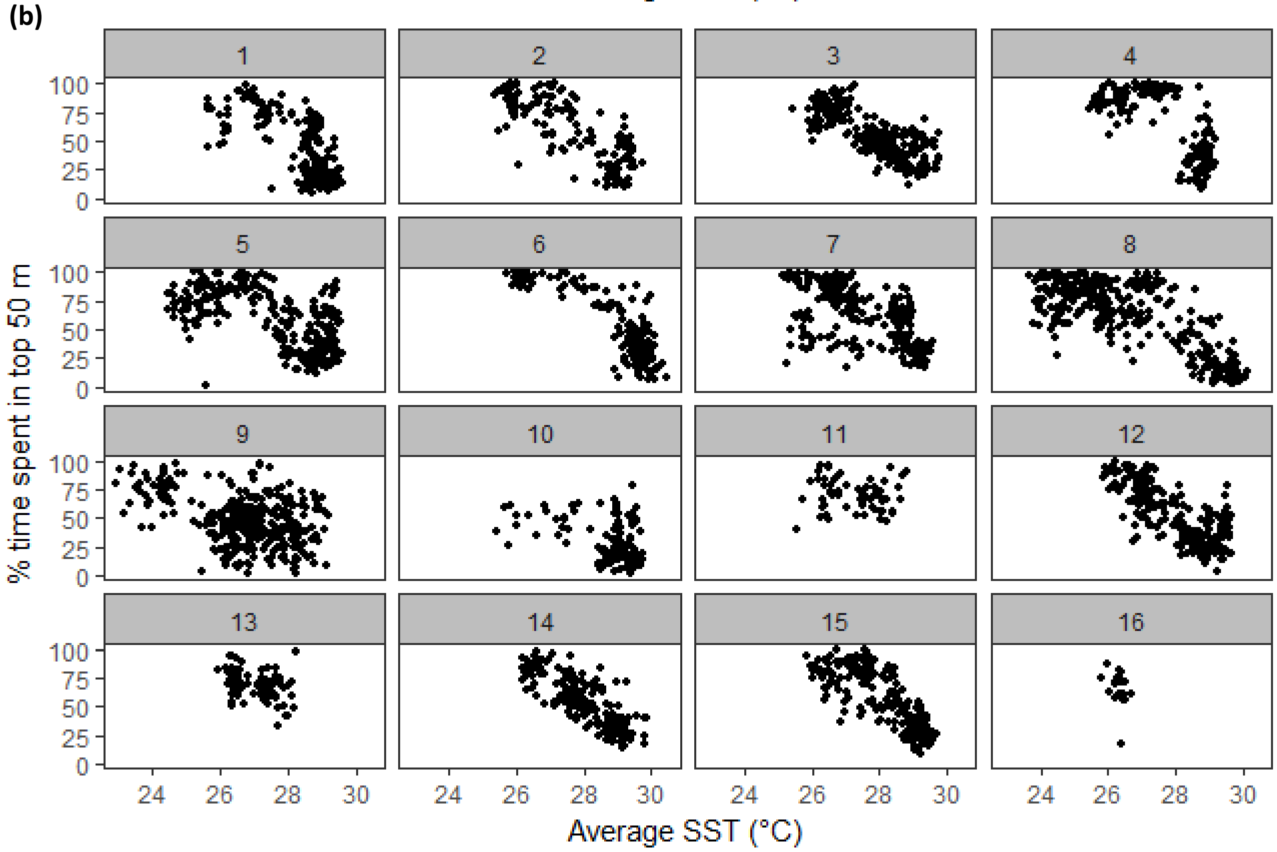

(c)

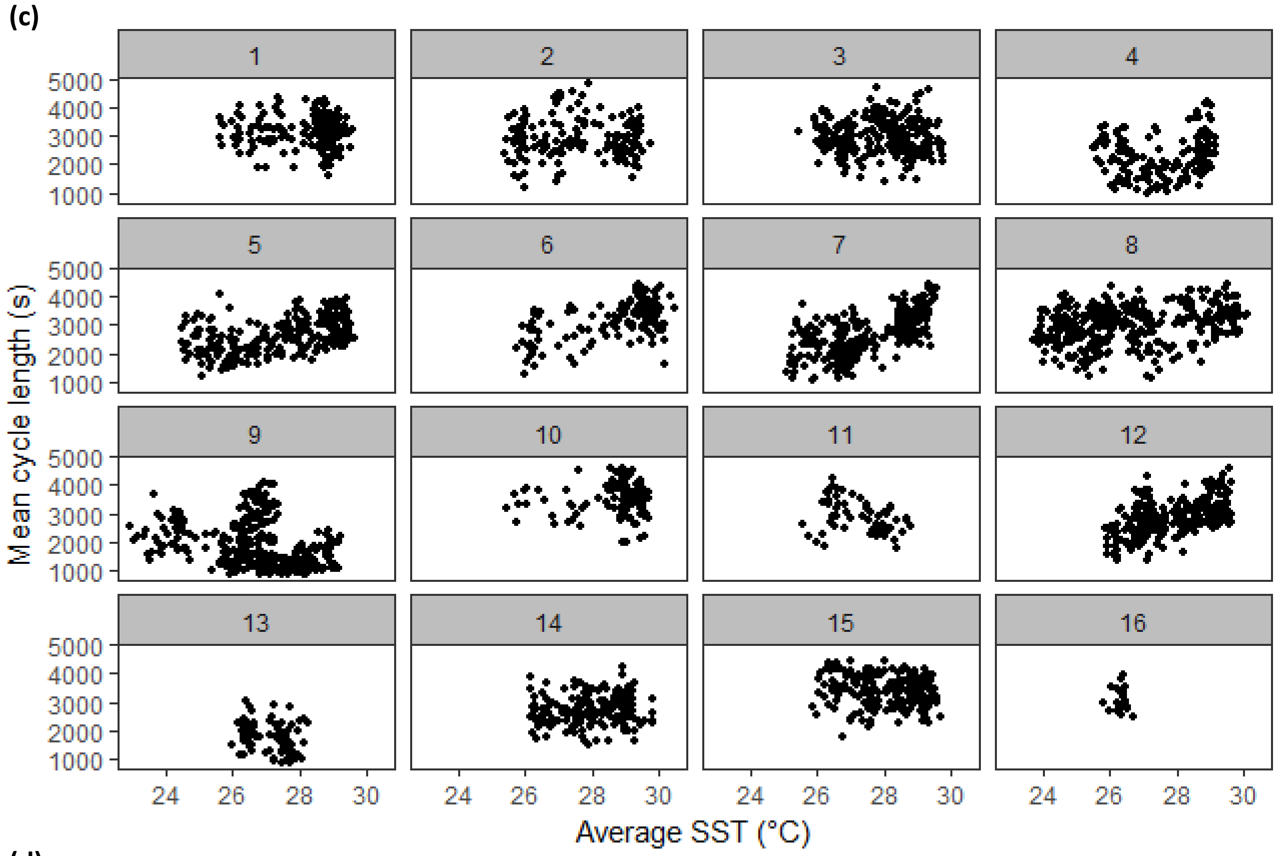
1
2
3
4
5
6
7
8
9
10
11
12
13
14
15
16
5000
4000
3000
2000
1000
Mean cycle length (s)
24
26
28
30
Average SST (°C)

(d)

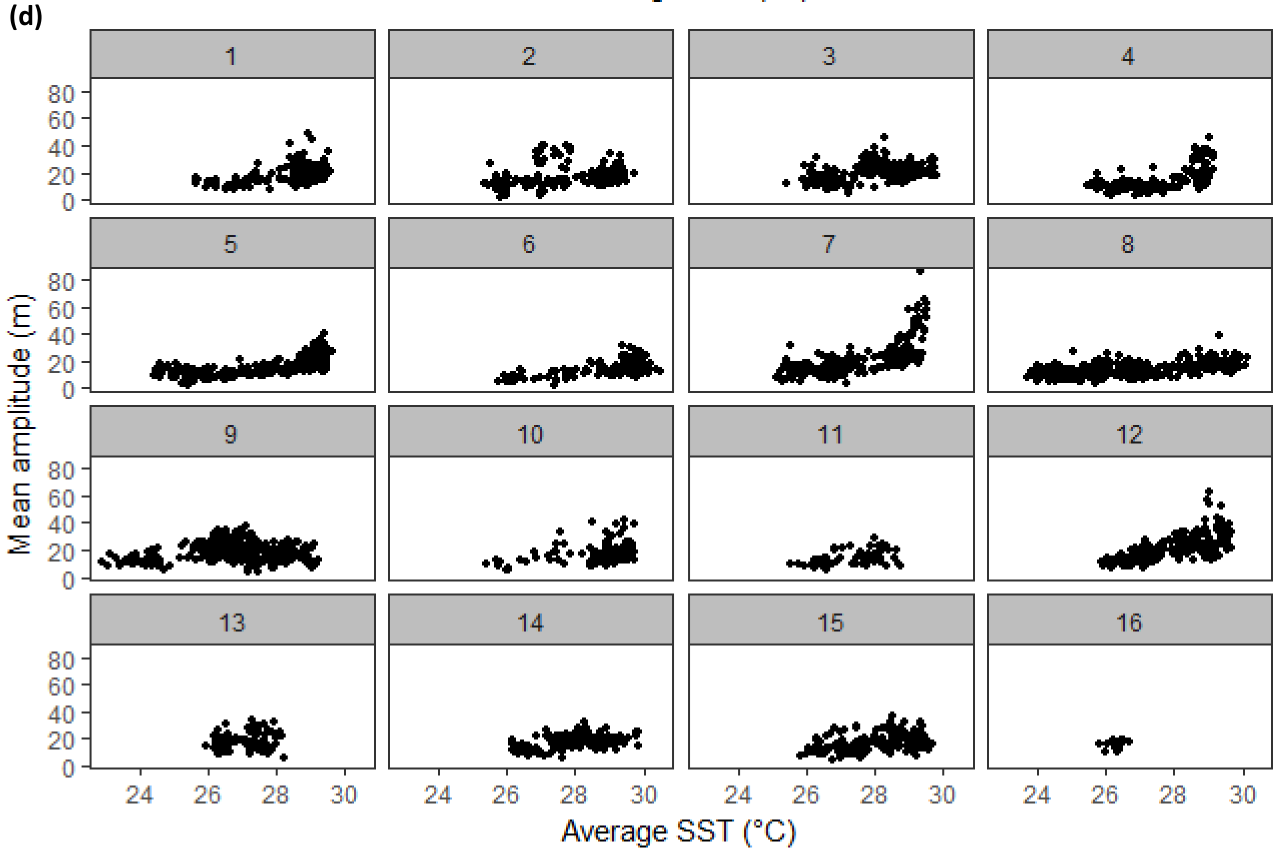
1
2
3
4
5
6
7
8
9
10
11
12
13
14
15
16
80
60
40
20
0
Mean amplitude (m)
24
26
28
30
Average SST (°C)
